# Exposure to environmental tobacco smoke (ETS) and risk of lung cancer in Montreal: a case–control study

**DOI:** 10.1186/1476-069X-12-112

**Published:** 2013-12-18

**Authors:** Mustafa Al-Zoughool, Javier Pintos, Lesley Richardson, Marie-Élise Parent, Parviz Ghadirian, Daniel Krewski, Jack Siemiatycki

**Affiliations:** 1 McLaughlin Center of Population Health Risk Assessment, University of Ottawa, Ottawa, Canada; 2 Centre de Recherche du CHUM, Université de Montréal, Montreal, Quebec, Canada; 3 INRS-Institut Armand-Frappier, Laval, Quebec, Canada

**Keywords:** Environmental tobacco smoke, Lung cancer, Case–control study

## Abstract

**Background:**

The objective of the present study was to examine the association between environmental tobacco smoke (ETS) and risk of lung cancer among never smokers, defined as subjects who smoked less than 100 cigarettes in their lifetime.

**Methods:**

We conducted a population-based case–control study on lung cancer in Montreal, Canada (1996–2000) including 1,203 cases and 1513 controls. The present analysis is restricted to the 44 cases and 436 population controls who reported never smoking and completed the questionnaire on lifetime ETS exposure. Collected information included duration and intensity of exposure from multiple sources: inside home (parents, spouses, roommates and any other co-resident) and outside homes (in vehicles, social settings, and workplace). Odds ratios (ORs) and 95% confidence intervals (CIs) were estimated between ETS and lung cancer, adjusting for age, sex, socioeconomic status (SES), and proxy respondent.

**Results:**

Overall there was no association between ETS cumulative exposure from all sources (measured in pack-years) and lung cancer: OR = 0.98 (95%CI: 0.40-2.38), comparing upper with lower tertiles of exposure. While there were no elevated ORs associated with ever having lived with parents who smoked (OR = 0.62; 95%CI: 0.32-1.21) or with spouses who smoked (OR = 0.39; 95%CI: 0.18-0.85), ETS exposure from sources outside homes was associated with a slight, although non-significant increased risk: OR = 2.30 (95%CI: 0.85-6.19) for the upper 50% exposed. There were no clear differences in ORs by age at exposure to ETS or by histologic type of tumour, though numbers of subjects in subgroup analyses were too small to provide reliable estimates.

**Conclusion:**

No clear association between lifetime ETS exposure from all sources and increased risk of lung cancer was found in the current study.

## Background

Less than 10% of all lung cancer cases occur among never smokers [1]. Environmental tobacco smoke (ETS) is one of the factors that have been linked to lung malignancies among these subjects. Several epidemiological studies conducted in North America, Asia, and Europe and two meta-analyses showed a slight increase in the risk of lung cancers among never smokers exposed to ETS, particularly among female non-smokers married to smokers [2,3], and the International Agency for Research on Cancer (IARC) concluded that there is sufficient evidence that ETS exposure causes lung cancer in humans [4,5]. Studies focused on sources of ETS other than spouses have also found a slight increased risk of lung cancer, such as those that examined the effect of ETS during childhood [6], in the workplace [7,8], or spouse, workplace and social sources [9]. But the available information is not definitive. Only a few have examined the effect of ETS exposure at different ages [10-12], and very few studies have looked at a host of possible sources of ETS. Most studies have involved very small sample sizes and because it is difficult to assemble large samples of nonsmoking lung cancer cases, it is necessary to increase the number of studies in order to improve the collective ability to discern patterns of risk.

Second-hand tobacco smoke is present in all places where smoking takes place: at home, in the workplace, in bars, restaurants, public buildings, and public transport [3]. The setting that represents the most important source of exposure varies by locale and time period, and it is very unlikely to find completely unexposed populations in urban areas.

In the late 1990s we carried out a population-based case–control study in Montreal, Canada, to examine the possible associations between hundreds of occupational substances and respiratory cancers. In addition to these factors, we set out to assess exposure to ETS among the nonsmoking cases and controls. The purpose was to examine the risk of developing lung cancer among never smokers as a consequence of ETS exposure from all sources combined: inside homes –parents, spouses, and other co-residents, as well from outside homes –workplace, social settings, and public and private transportation. In addition, we examined the effect of exposure at different life stages.

## Methods

### The Montreal study

A case–control study of lung cancer was conducted from 1996 to 2000 in Montreal and its surrounding suburbs, an area comprising a population of 3.1 million in 1996. It included males and females aged 35–75 years of age who were Canadian citizens and residents of the study area. Incident cases occuring in 1996 and 1997 were ascertained in the 18 largest hospitals located in the metropolitan area, and identified through hospital tumor registries or through active monitoring of pathology department records. A total of 1203 lung cancer cases were interviewed, representing a response rate of 86%. Controls were randomly sampled from population based electoral lists, stratified by sex and age to represent the distribution of cases. A total of 1513 controls were interviewed (a response rate of 70%) from 1996 until early 2000. Detailed information was collected on socio-demographic characteristics, residential history, medical history, smoking, diet, and detailed occupational history. Further details about the study can be found elsewhere [13,14].

### Subjects in the current study

Only never smokers, defined as those subjects who reported smoking less than 100 cigarettes during their lifetime, were selected for the current study. Those subjects who reported never smoking tobacco completed a section on exposure to ETS. A total of 480 subjects, 44 cases and 436 controls met this criterion and were included in this study. For some of the subjects, part of the whole interview was answered by a proxy respondent, usually the spouse (Table 1). It is likely that some of the information provided by proxies was not reliable, such as ETS exposure during childhood or in the workplaces. For this reason we present results including and excluding proxy respondents.

**Table 1:** Distribution of subjects according to selected socio-demographic characteristics

		**All Subjects**				**self-respondents only**			
	**Categories**	**Controls**		**Cases**		**Controls**		**Cases**	
		**N=436**	**%**	**N=44**	**%**	**N=410**	**%**	**N=32**	**%**
Sex									
	Male	128	29%	13	30%	117	29%	10	31%
	Female	308	71%	31	70%	293	71%	22	69%
Age									
	<= 55	92	21%	12	27%	89	22%	7	22%
	56-65	136	31%	9	20%	133	32%	9	28%
	66-75	208	48%	23	52%	188	46%	16	50%
Ethnicity									
	French Canadian	246	56%	25	57%	239	58%	17	53%
	Other	190	44%	19	43%	171	42%	15	47%
Schooling									
	< 7 years	137	31%	16	36%	123	30%	10	31%
	7-12 years	172	39%	18	41%	163	40%	14	44%
	13+ years	127	29%	10	23%	124	30%	8	25%
Family income*									
	< 30.000	140	32%	17	39%	133	32%	10	31%
	30.000 - 45.000	172	39%	15	34%	161	39%	12	38%
	> 45.000	124	28%	12	27%	116	28%	10	31%
Respondent									
	Self	410	94%	32	73%	410	100%	32	100%
	Proxy	26	6%	12	27%				

*Median family income for census tract in Can$.

### Assessment of ETS exposure

This section of the personal interview was primarily designed to assess exposure to ETS during different life periods and from different sources at home (from parents or from other co-inhabitants including spouses and/or roommates), in vehicles (such as public transportation, taxis, or cars), in social settings (bars, restaurants), and at workplaces. The following information was elicited to evaluate duration and intensity of ETS exposure at homes: years of exposure, hours per day of exposure, age period when subject was exposed, and number of cigarettes/day of ETS exposure. Information for vehicles, social settings, and workplace included the duration of exposure (in years), number of hours per week of exposure, and exposure intensity at the source (slight, moderate, or heavy).

### Cumulative indices of ETS and statistical analyses

Exposure to ETS was analyzed as a dichotomous variable (exposed or unexposed) and as a quantitative variable. Three indices were computed to summarize lifetime ETS exposure: i) person-years of exposure, defined as the number of years spent in homes with ETS multiplied by the number of smokers; ii) duration of exposure (hours/day x years) calculated as the product of the average number of hours/day of ETS exposure and the number of years of exposure; and iii) cumulative exposure, defined as the total number of pack-years of lifetime ETS exposure. Ordinal categories of ETS intensity in vehicles, public places, and work (mild, moderate, and heavy) were converted into number of cigarettes per day according to the following conversion algorithm: 5, 15, and 25 cigarettes/day, for mild, moderate, and heavy, respectively. This was roughly based on smoking patterns in North America in the 1950s and 1960s [15]. All of these indices were computed for lifetime ETS exposure, as well as for two specific life periods: up to and after 20 years of age. It should be emphasized that these various indices represent the amount of smoking that was being done by other people in the proximity of the subject; there is no attempt to translate these indices into equivalences in terms of active smoking.

Unconditional logistic regression models were fitted to estimate the odds ratios (ORs), and their 95% confidence intervals (CIs), of lung cancer associated with ETS exposure from different sources. Regression models included potential confounders such as sex, age, education level, and median household income of the census tract of residence. Inclusion of a variable representing ever exposure to known occupational lung carcinogens did not modify estimates; hence it was not included in the final model. We defined as lung carcinogens those chemicals classified by IARC as Group 1 (carcinogenic to humans) or Group 2A (probably carcinogenic to humans) [16], including asbestos, crystaline sillica, chromium VI compounds, arsenic compounds, cadmium compounds, benzo(a)pyrene, and/or diesel engine emissions. All statistical analyses were two-sided at 0.05 significance level and were conducted using SAS software version 9.2 (SAS Institute, Cary, NC).

Ethical approval was obtained from ethics committees of each of the 18 participating hospitals involved in the original research, and from institutional review boards of participating universities (McGill University and Université de Montréal). All participating subjects provided informed consent.

## Results

A total of 44 never-smoking cases and 436 never-smoking controls were included in the present analysis. There was a similar distribution by age and sex between cases and controls (Table 1). A higher proportion (27%) of proxy interviews were conducted for cases than for controls (6%). Controls had on average more years of formal education than cases; 29% reached postsecondary school compared to 23% of cases. Median census tract home income was similar for cases and controls. More than half of the cases were diagnosed as adenocarcinomas.

Table 2 shows OR estimates for lung cancer according to ETS exposure from parents. Most of the exposure occured at an early age (before 20 years of age). Ever having lived with a smoker parent did not increase the risk of lung cancer. Neither were duration of exposure (measured in parent-years and in hours/day × years) or cumulative exposure (measured in pack-years), associated with lung cancer. All point estimates were below 1.0, although none of them reached statistical significance. Results including all subjects or only self-respondents did not differ appreciably. Table 2 also shows ORs in relation to spousal smoking status. Having lived with a smoking spouse did not increase the risk of lung cancer. Paradoxically, our results showed a significant inverse association (OR = 0.39; 95%CI: 0.16-0.85), which did not change when restricting the analysis to self-respondents only. We were not able to analyze different subcategories of spousal smoking due to small numbers of ever smoking spouses in this population.

**Table 2:** Odds Ratios (ORs) of lung cancer associated with ETS from parents or spouses

			**All subjects**					**Self-respondents only**				
**ETS source**			**Controls**	**Cases**				**Controls**	**Cases**			
		**Categories**	**N=436**	**N=44**	OR*	**95%CI**		**N=410**	**N=32**	OR*	**95%CI**	
**Parents**												
	Never		195	26	1	(ref.)		179	17	1	(ref.)	
	Ever		241	18	0.62	0.32	1.21	231	15	0.69	0.33	1.43
	Person-years											
		1-21 parent-yrs	135	10	0.62	0.28	1.36	129	8	0.65	0.27	1.57
		22+ parent-yrs	106	8	0.64	0.27	1.50	102	7	0.73	0.29	1.84
	Duration											
		<=75 (hrs/d x yrs)	119	9	0.64	0.28	1.46	114	8	0.74	0.31	1.79
		>75 (hrs/d x yrs)	122	9	0.61	0.27	1.38	117	7	0.63	0.25	1.59
	Cumulative exposure											
		<=3 pack-years	146	9	0.50	0.22	1.13	137	8	0.63	0.26	1.50
		>3 pack-years	95	9	0.86	0.37	1.99	94	7	0.79	0.31	2.02
**Spouses**												
	Never		262	34	1	(ref.)		242	25	1	(ref.)	
	Ever		174	10	0.39	0.18	0.85	168	7	0.37	0.15	0.93

*Adjusted for sex, age, median income, and proxy (if applicable).

Table 3 shows the OR estimates for exposure inside homes, including ETS from all co-residents combined: parents and other relatives, spouses, roommates, and any other people having shared the household with the index subjects. Similar to results for parents only or spouses only, we did not find an increased risk of lung cancer for those subjects ever exposed to ETS from all co-residents, in relation to either duration of exposure or cumulative exposure. Moreover, all point estimates were below 1.0, although none of them reached statistical significance. There was no clear difference in OR according to whether exposure occurred in childhood or adulthood (Table 3).

**Table 3:** Odds Ratios (ORs) of lung cancer associated with ETS from inside homes (all co-residents combined)

			**All subjects**					**Self-respondents only**				
**ETS from co-residents**			**Controls**	**Cases**				**Controls**	**Cases**			
		**Categories**	**N=436**	**N=44**	OR*	**95%CI**		**N=410**	**N=32**	OR*	**95%CI**	
	Never		113	16	1	(ref.)		105	11	1	(ref.)	
	Ever		323	28	0.63	0.32	1.25	305	21	0.66	0.30	1.46
	Person-years											
		1-21 coresident-yrs	95	12	0.93	0.40	2.12	90	9	0.95	0.37	2.44
		22+ coresident-yrs	228	16	0.50	0.23	1.08	215	12	0.53	0.22	1.28
	Duration											
		<=100 (hrs/d x yrs)	126	13	0.75	0.34	1.69	118	11	0.89	0.36	2.19
		>100 (hrs/d x yrs)	197	15	0.54	0.25	1.20	187	10	0.51	0.20	1.27
	Cumulative exposure											
		<=4 pack-yrs	150	14	0.67	0.31	1.48	140	11	0.76	0.31	1.86
		>4 pack-yrs	173	14	0.60	0.27	1.34	165	10	0.58	0.23	1.47
	Exposure only < 20 yrs age		57	8	1.09	0.42	2.83	54	7	1.24	0.44	3.51
	Exposure only >= 20 yrs age		56	6	0.72	0.26	2.06	53	3	0.52	0.14	2.02
	Exposure both < & >= age 20		210	14	0.48	0.22	1.05	198	11	0.52	0.21	1.29

*Adjusted for sex, age, median income, and proxy (if applicable).

In Table 4 we present results according to ETS exposure from sources outside home: vehicles, public places such as bars and restaurants, and the occupational environment. In contrast with ETS exposure sources in the household (Tables 2 and 3), there was an increased risk of lung cancer, although non-significant, in relation to ETS exposure in these outside-the-home venues.

**Table 4:** Odds Ratios (ORs) of lung cancer associated with ETS from outside homes (vehicle, public places, work)

		**All subjects**					**Self-respondents only**				
		**Controls**	**Cases**				**Controls**	**Cases**			
	**Categories**	**N=436**	**N=44**	OR*	**95%CI**		**N=410**	**N=32**	OR*	**95%CI**	
	Never	175	16	1	(ref.)		160	8	1	(ref.)	
	Ever	261	28	1.51	0.75	3.05	250	24	1.95	0.84	4.54
Duration											
	<= 64 (hrs/d x yrs)	146	16	1.47	0.68	3.19	141	12	1.74	0.69	4.42
	>64 (hrs/d x yrs)	115	12	1.57	0.65	3.81	109	12	2.30	0.85	6.19
Duration											
	<=1 pack-year	124	16	1.70	0.78	3.69	119	12	2.05	0.81	5.22
	>1 pack-year	137	12	1.28	0.54	3.03	131	12	1.84	0.70	4.85

*Adjusted for sex, age, median income, and proxy (if applicable).

Table 5 shows OR estimates associated with ETS exposure from all sources combined: inside homes (parents, siblings, other relatives, spouses, and/or any other co-residents), and outside homes (vehicles, public places, and the work environment). Not surprisingly, it was not possible to create a ‘non exposed’ category, considering that most subjects were exposed to ETS in at least one milieu; for instance, among self-respondents, only two cases and 44 controls reported never experiencing ETS. For this reason we created categories according to tertiles of the distribution of ETS duration (hours/day × years) and ETS cumulative exposure (pack-years) among self-respondent cases. We assumed equal weights for cigarettes and pack-years irrespective of the type of exposure. While the highest tertiles of exposure to ETS showed no increased in risks compared with the lowest, the middle tertiles showed some elevations in point estimates of ORs, albeit not statistically significant.

**Table 5:** Odds Ratios (ORs) of lung cancer associated with ETS from all sources combined

		**All subjects**					**Self-respondents only**				
		**Controls**	**Cases**				**Controls**	**Cases**			
**Index**		**N=436**	**N=44**	OR*	**95%CI**		**N=410**	**N=32**	OR*	**95%CI**	
Duration (in hrs/d x yrs)											
	<=100	173	18	1	(ref.)		163	11	1	(ref.)	
	101-190	103	13	1.19	0.55	2.60	94	11	1.74	0.73	4.19
	>190	160	13	0.86	0.40	1.85	153	10	0.97	0.40	2.36
Cumulative exposure (in pack-years)											
	<=2.5	176	18	1	(ref.)		163	11	1	(ref.)	
	2.5-6	101	12	1.37	0.61	3.06	96	11	1.68	0.70	4.04
	>6	159	14	0.96	0.45	2.04	151	10	0.98	0.40	2.38

*Adjusted for sex, age, median income, and proxy (if applicable).

While there were not sufficient numbers of cases of specific histologic subtypes to warrant presentation of detailed analyses by subtype, we did carry out analyses in particular for adenocarcinoma of the lung. The results were not noticeably different from those shown for all histologic types combined. For instance, combining all sources of ETS and comparing the top tertile of pack-years with the lowest tertile (as per Table 5) gave an OR of 0.95 (95%CI 0.43-2.29).

## Discussion

There have been several studies investigating the association between ETS and lung cancer [2-6]. While a consensus has developed that ETS is a risk factor for lung cancer, in fact results from studies conducted to date have been very heterogeneous [2], and further exploration of this hypothesis remains an important one to explore, both overall and in terms of characterizing the possible risks by type of exposure to ETS and type of lung cancer. Because of the difficulty of assembling a large series of nonsmoking lung cancer cases in one locale, except for large international collaborations, most studies have been small, including our own. But it is also a reason for producing and publishing as many valid studies as possible, so as to provide the basis for future syntheses and meta-analyses.

The study on which this analysis was based was designed primarily to examine the risk of lung cancer related to occupational exposures. As in any case–control study of lung cancer, the proportion of cases who were lifelong nonsmokers was very low, and even though the total number of cases in our study was over 1200, the number of never smoking cases was only 44. This is the main limitation of this study, although this is somewhat palliated by the fact that exposure to ETS is quite prevalent. As a result of the low numbers, the estimates of OR from our analysis alone are quite imprecise. But this is typical of most studies of ETS and cancer. We believe that our results are useful, not only because they stand by themselves, but because they may be included in future meta-analyses or pooled analyses, which may accumulate sufficient numbers of subjects to achieve more precise results. In addition, to our knowledge, there is no other published study assessing as many sources of ETS as the present manuscript.

In the present manuscript we present results for ETS exposure from individual sources, namely parents, spouses, other co-residents, and outside home sources. Results for ETS exposure outside homes were associated with higher risk than for exposure inside homes. This is in accordance with the several studies that reported higher intensity and more consistent exposure in the workplace, an exposure that occurs outside homes. From risk assessment perspective, the results presented in Table 5 for TES exposure from all sources are considered the most relevant. Also it is not surprising that combining all sources of ETS, very few subjects reported never have been exposed, especially in a population that was mostly born before 1960 and grew up in a society where smoking was very common.

Strengths of this work include the detailed information collected among never smokers, including ETS exposure from several different sources and during different life stages. In addition, we adjusted the analyses for several potential confounders, including exposure to occupational carcinogens and socioeconomic status. Control for socioeconomic status is particularly important since some studies suggested that subjects with lower socioeconomic status have more intense and longer workplace exposure, which results in higher cumulative lifetime ETS exposure [7,8]. Our study also looked into the effects of increasing levels of combined household and workplace exposures, which better represents total ETS exposure. Limitations of our study include the already cited low number of cases, and exposure misclassification that inevitably occurred in attempting to retrospectively estimate whether or not each subject was exposed, and the magnitude of this exposure. It is very likely that subjects who reported never exposure to ETS had indeed been exposed. However, it is also likely that their cumulative level of exposure is low, and they served well as a reference group for those subjects with high levels of exposure.

We failed to find a significant association between ETS and lung cancer. Possible explanations include the small sample size and the small ETS exposure levels. Measurement bias resulting from inaccuracies in the assessment of individual exposure to ETS may lead to error in estimation of the risk. Subjects may not recall all exposure sources especially outside the home. For example, it was reported that for 25-50% of women who work outside the home, the spouse’s smoking habit may not accurately reflect their overall exposure to ETS [17]. Similarly, controls who report no ETS exposure may in fact be exposed to other sources in the workplace or the public. If this misclassification of exposure is equal among cases and controls it will lead to underestimation of the risk [18,19].

Regarding ETS exposure inside homes, which mainly occurs at younger ages, we did not find a significant increased risk of lung cancer. These findings are not in disagreement with previous investigations. Some of the largest studies did not find an association between exposure during childhood and risk of lung cancer [11,12,20,21], including a multi-center study reporting that ETS exposure during childhood was associated with reduced risk of lung cancer: OR = 0.78 (95%CI 0.64-.96) [7]. On the other hand, recent investigations have shown an increased risk of lung cancer associated with ETS exposure during childhood. In a large cohort study conducted in 10 European countries (European Prospective Investigation into Cancer and Nutrition, EPIC), it was estimated that the hazard ratio (HR) for lung cancer risk from ETS exposure during childhood was 2.00 (0.94–4.28); among children with daily exposure for many hours each day the hazard ratio was 3.63 (1.19–11.12) [6].

Other studies have also reported increased of lung cancer associated with ETS exposure at younger ages [10,22]. Recent investigations that examined ETS during adulthood have also shown an increased risk of lung cancer. A meta-analysis estimated an OR of 1.27 (95%CI: 1.17-1.37) in the risk of lung cancer among non-smoking women exposed to spousal ETS [4]. A second meta-analysis showed a relative risk of lung cancer among nonsmoking women ever exposed to ETS from their husbands of 1.20 (95%CI: 1.12-1.29) [5]. Studies that focused on other sources of ETS have also found a slight increased risk of lung cancer, such as in the workplace [7,8], or combining spouse, workplace, and social sources [9].

We conducted several exploratory analyses, including comparing risk in groups younger than 65 years of age at diagnosis versus older than 65, males versus females, and risk due to exposure at work only. None of these analyses resulted in increased risk. In a recent meta-analysis of 22 studies, ETS exposure in the workplace was linked to increased risk of lung cancer (OR = 1.24, 95%CI: 1.18-1.29) [23]. In the EPIC cohort, workplace exposure to ETS was linked to lung cancer among women: HR of 2.13 (1.6–3.4) [24]. Despite not detecting risk due to ETS exposure at work (data not shown), our results suggest an increased risk of lung cancer for subjects who were exposed from outside the home.

A large proportion of the cases (52%) in our study were adenocarcinoma. In our analyses, based on small numbers, there was no noticeable difference in ETS ORs for adenocarcinoma and for all tumour types combined. Evidence from previous literature is conflicting. Several studies reporting results on different histological types of lung cancer found that risk of other types was higher than that of adenocarcinoma [7,12,20,21,25,26], some found no differences by histologic type [27,28], while another study reported higher risk for adenocarcinomas [8].

Regardless of the effect on lung cancer, ETS exposure is far from harmless and it should be reduced and controlled. It contributes to significant morbidity and mortality among children, including preterm birth, intrauterine growth retardation, perinatal mortality, respiratory illness, neurobehavioral problems, and decreased performance in school [29].

## Conclusions

We did not find a clear association between lifetime ETS exposure from all sources and increased risk of lung cancer. Among the sources of ETS that were examined, only exposure outside the home, in adulthood, showed any indication of excess risks. Small numbers preclude strong inferences from this study on its own.

### Consent

A Written informed consent was obtained from all participating subjects for the publication of this report.

## Competing interests

The authors declare that they have no competing interests.

## Authors’ contributions

MA carried out the main analyses and drafted the manuscript. JP participated in the analysis of the data and wrote sections of the manuscript. LR participated in the study design, MP contributed to the conception of the original study. PG contributed in the study design, DK participated in the design and coordination. JS conceived the study, participated in its design and coordination, and edited the manuscript. All authors read and approved the final manuscript.
